# Application of Loop-Mediated Isothermal Amplification (LAMP) Assay for Detection of *Leishmania infantum* Strain from Brazil

**Published:** 2020

**Authors:** Gilberto SILVA NUNES BEZERRA, Walter Lins BARBOSA JÚNIOR, Nilma CINTRA LEAL, Zulma Maria DE MEDEIROS

**Affiliations:** 1. Postgraduate Program in Health Sciences, Universidade de Pernambuco (UPE), 50100-130 Recife, Pernambuco, Brazil; 2. Department of Parasitology, Instituto Aggeu Magalhães (IAM), Fundação Oswaldo Cruz (FIOCRUZ), 50670-420 Recife, Pernambuco, Brazil; 3. Department of Microbiology, Instituto Aggeu Magalhães (IAM), Fundação Oswaldo Cruz (FIOCRUZ), 50670-420 Recife, Pernambuco, Brazil

## Dear Editor-in-Chief

Visceral Leishmaniasis (VL), also known as *Kala-azar*, is a life-threatening disease responsible for 300.000 new cases per year, more than 90% of them occurring in Bangladesh, Brazil, Ethiopia, India, Nepal, South Sudan and Sudan (1). The *L. donovani* complex is responsible for the worldwide burden of VL represented by *L. donovani* in East Africa and Indian sub-continent followed by *L. infantum* in Europe, North Africa and Latin America (2). Since both conventional techniques (parasitological and serological) have several limitations for VL diagnosis, scientists have explored the field of molecular biology for nucleic acid amplification tests (3). LAMP (Loop-Mediated Isothermal Amplification) is a novel nucleic acid amplification method emerged as a promising diagnostic tool for VL diagnosis amplifying a target DNA sequence with high sensitivity, specificity and efficiency under isothermal conditions (4).

We evaluated the application of a LAMP system developed in the Old World using a *L. infantum* reference strain from Brazil, expecting this protocol may contribute to reducing the VL incidence rate in South America (5).

We performed a BLASTn (*Basic Local Alignment Search nucleotide*-https://blast.ncbi.nlm.nih.gov/Blast.cgi) using the same *L. infantum* kDNA sequence applied to primers design (GenBank accession number Z35271). The alignment result demonstrated sequences with significant homology to *L. infantum* (over 97.86% of identity) such as *L. donovani* and *L. chagasi*. Hence, we used standard genomic DNA from *Leishmania (L.) infantum chagasi* IOC-L 3328 (MHOM/BR/2011/COS) to prepare a dilution series standard DNA (10 ng – 0.01 fg) to evaluate the limit of detection for Polymerase Chain Reaction (PCR) and LAMP system. PCR assay was performed applying the protocol developed by Gualda et al (6) using primers FLC2 (5′-GTCAGTGTCGGAAACTAATCCGC-3′) and RLC2 (5′-GGGAAATTGGCCTCCCTGAG-3′), which amplification product was 230bp.

**Fig. 1:**
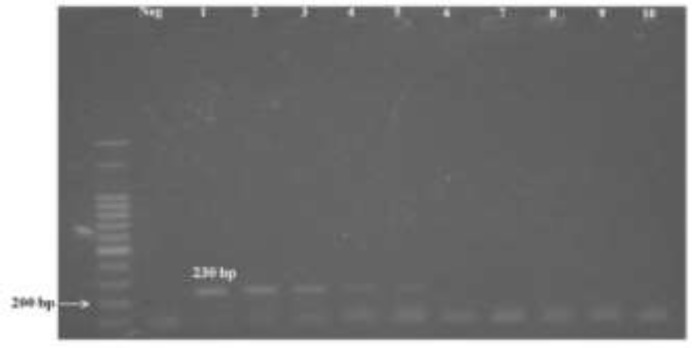
PCR results are displayed in 1.5% agarose gel staining with ethidium bromide visualized under UV light. The 10-fold dilution curve is constituted by: Molecular marker of 100 bp, Neg – Control negative, (1) 10ng, (2) 1ng, (3) 100pg, (4) 10pg, (5) 1pg, (6) 100fg, (7) 10fg, (8) 1fg, (9) 0.1fg and (10) 0.01fg

**Fig. 2:**
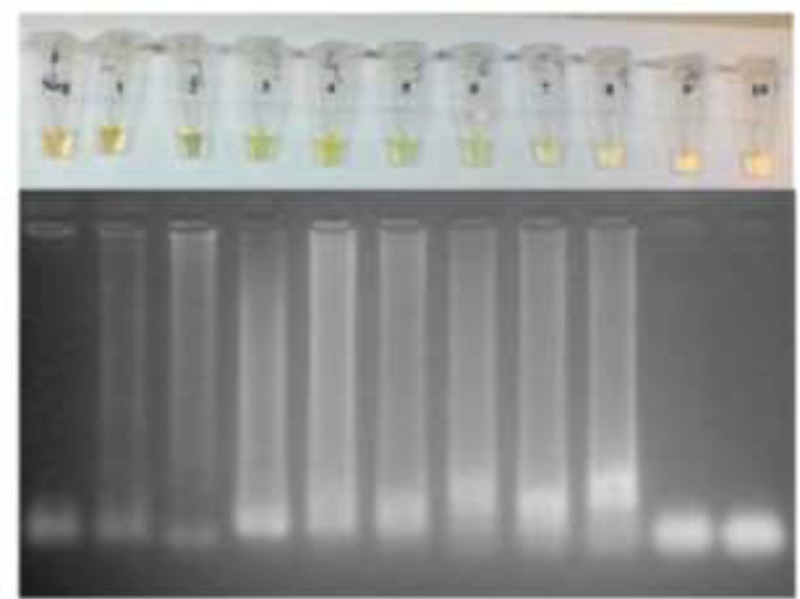
LAMP results in 2% agarose gel staining with ethidium bromide visualized under UV light. The 10-fold dilution curve is constituted by: Neg – Control negative, (1) 10ng, (2) 1ng, (3) 100pg, (4) 10pg, (5) 1pg, (6) 100fg, (7) 10fg, (8) 1fg, (9) 0.1fg and (10) 0.01fg. On top is seen LAMP reaction tubes after addition of SYBR Green I

Figure 1 shows FLC2 and RLC2 limit of detection, achieving a maximum analytical sensitivity of 1 pg. In addition, it was applied the isothermal amplification protocol in our dilution series standard DNA. In Fig. 2, LAMP results could be interpreted as suggestive of nucleic acid amplification-based on changing of color and electrophoresis profile with analytical sensitivity of 1 fg, but after trying to optimize the system several times, changing magnesium concentration (2, 4, 6 and 8mM) and temperature (52, 55, 58, 61 and 64 °C), the results were not reproducible.

Thus, there is a notorious effort from the scientific community to identify accurate and sensitive methods for diagnosing VL, which means an investment of knowledge, money, time and technical support. In Bangladesh, after develop the first LAMP system for VL diagnosis (7), a validation study was performed from this first LAMP assay with minor modifications, achieving a better clinical sensitivity performance (8). In India, also a validation study was performed on the first isothermal amplification system for VL diagnosis (9). Despite they achieved great sensitivity and specificity results, the LAMP assay was found less sensitive for strains of *L. (L.) donovani* originating from distinct geographical regions other than India.

Therefore, there is a meaningful point taken into consideration before trying to apply any LAMP protocol for VL diagnosis in different endemic settings, which would be the inter- and intragenic diversities of *Leishmania* species. Finally, we do not recommend the application of the LAMP protocol that we evaluated for VL studies in Brazil and South America based on reproducibility limitations.
